# Development of a behavioural framework for dementia care partners’ fall risk management

**DOI:** 10.1186/s12877-022-03620-4

**Published:** 2022-12-17

**Authors:** Yuanjin Zhou, Clara Berridge, Nancy R. Hooyman, Tatiana Sadak, Tracy M. Mroz, Elizabeth A. Phelan

**Affiliations:** 1 Steve Hicks School of Social Work, University of Texas at Austin, Austin, USA; 2 School of Social Work, University of Washington, Seattle, USA; 3 School of Nursing, University of Washington, Seattle, USA; 4 Department of Rehabilitation Medicine, University of Washington, Seattle, USA; 5 School of Medicine, Division of Gerontology and Geriatric Medicine, School of Public Health, Department of Health Systems and Population Health, University of Washington, Seattle, USA

**Keywords:** Cognitive impairment, Dementia, Fall prevention, Caregiving, Fall risk, Health behaviours

## Abstract

**Background:**

Although older adults living with dementia (OLWD) are at high risk for falls, few strategies that effectively reduce falls among OLWD have been identified. Dementia care partners (hereinafter referred to as “care partners”) may have a critical role in fall risk management (FRM). However, little is known about the ways care partners behave that may be relevant to FRM and how to effectively engage them in FRM.

**Methods:**

Semi-structured, in-depth interviews were conducted with 14 primary care partners (age: 48–87; 79% women; 50% spouses/partners; 64% completed college; 21% people of colour) of community-dwelling OLWD to examine their FRM behaviours, and their observations of behaviours adopted by other care partners who were secondary in the caring role.

**Results:**

The analysis of interview data suggested a novel behavioural framework that consisted of eight domains of FRM behaviours adopted across four stages. The domains of FRM behaviours were 1. functional mobility assistance, 2. assessing and addressing health conditions, 3. health promotion support, 4. safety supervision, 5. modification of the physical environment, 6. receiving, seeking, and coordinating care, 7. learning, and 8. self-adjustment. Four stages of FRM included 1. supporting before dementia onset, 2. preventing falls, 3. preparing to respond to falls, and 4. responding to falls. FRM behaviours varied by the care partners’ caring responsibilities. Primary care partners engaged in behaviours from all eight behavioural domains; they often provided functional mobility assistance, safety supervision, and modification of the physical environment for managing fall risk. They also adopted behaviours of assessing and addressing health conditions, health promotion support, and receiving, seeking and coordinating care without realizing their relevance to FRM. Secondary care partners were reported to assist in health promotion support, safety supervision, modification of the physical environment, and receiving, seeking, and coordinating care.

**Conclusions:**

The multi-domain and multi-stage framework derived from this study can inform the development of tools and interventions to effectively engage care partners in managing fall risk for community-dwelling OLWD.

**Supplementary Information:**

The online version contains supplementary material available at 10.1186/s12877-022-03620-4.

## Background

Falls are among the most prevalent and debilitating health issues that hinder older adults’ capability to age in place [1, 2]. Older adults living with dementia (OLWD) experience greatly heightened fall risk compared to their age-matched peers without dementia [3] due to various biological, cognitive, and behavioural risk factors [4]. They are also more likely to be injured [5], less likely to recover [6], and have a higher rate of institutionalization [7], and mortality [8]. Falls are the second leading cause of hospital readmission for OLWD [9]. Despite the severity of this issue, evidence-based fall prevention programs for community-dwelling OLWD are limited [10], pointing to the pressing need for feasible and effective strategies to address the risk of falling among this population.

Internationally, most OLWD at home are cared for by informal care partners (e.g., family, partners, friends) [11–13]. Previous studies suggested that supporting care partners to manage fall risk might be effective in reducing this risk for community-dwelling OLWD [14]. A recent systematic review found that care partners adopted multi-level (individual, interpersonal, physical environment, and community/institutional level) strategies to manage fall risk for OLWD that they cared for; however, these review findings were based on studies that were not designed to systematically examine care partners’ experiences of fall risk management (FRM) [15]. Available tools and interventions developed to support or involve care partners in managing fall risk for OLWD included a decision-making discussion tool for the falls prevention [16], a Home Safety Toolkit [17], and a dyadic Tai Chi exercise program [18]. However, none of these studies examined the impact of interventions on care partners’ adoptions of FRM strategies and if these strategies effectively mitigated OLWD’s risk of falling.

Furthermore, previous studies suggested that community-dwelling OLWD who had care support often had more than one care partner [19]. Although all the care partners might contribute to care activities, one care partner is typically the primary source of care and others might take on secondary care responsibilities [20, 21]. Previous studies suggest that care responsibilities might differ between primary and secondary care partners of OLWD [22, 23]. Primary care partners tend to provide practically round-the-clock care, including OLWD’s activities of daily living, mobility, instrumental activities of daily living, and overall health management; secondary care partners often provide supplementary support to OLWD, especially supporting OLWD’s basic daily tasks [22–25]. However, previous studies have not described how care partners’ FRM behaviours differ by their caring responsibilities [15], which might not give a complete picture of FRM in the context of dementia caregiving.

These gaps suggested the need to develop a comprehensive framework of dementia care partners’ FRM based on their lived experiences to guide clinical assessment and intervention development. The present study used in-depth, semi-structured interviews to develop such a framework by addressing the research question – “How do care partners with different care responsibilities manage fall risk for community-dwelling OLWD?”.

## Methods

### Study design

Semi-structured, in-depth interviews were used to systematically explore care partners’ experiences in FRM. We drew from methods of the Informed Grounded Theory approach developed by Thornberg (2012) to allow researchers to explicitly incorporate knowledge from extant literature into an interview guide [26]. This approach will allow researchers to generate theory founded in data by grounded theory strategies while also being informed by existing research and theoretical frameworks “in a sensitive, creative, and flexible way instead of seeing them as obstacles and threats” [26]. To generate a meaningful and innovative theoretical framework for describing dementia care partners’ experiences of FRM and to strengthen our theoretical sensitivity in data collection and analysis, we utilized different theoretical perspectives and relevant literature on dementia caregiving and FRM [26].

Based on the theory of symbolic interactionism [27], we utilized data analytic strategies from this grounded theory approach to delve deeply into different care partners’ experiences of interacting with OLWD and the context of caregiving to manage OLWD’s risk of falling [28]. These strategies allowed the production of knowledge and understanding that could be applied to the intervention development [29]. Techniques of this grounded theory approach that were used and discussed below included theoretical sampling, coding procedures, constant comparative methods, and memo writing to increase the credibility and the consistency of the findings and the framework that was developed [28]. The qualitative methods and reporting of results adhered to the Consolidated Criteria for Reporting Qualitative Studies (COREQ) [30]. The Institutional Review Board at the University of Washington approved this research (UW IRB # STUDY00007327).

### Use of literature to strengthen theoretical sensitivity

The literature on fall risk characterized falls’ consequences, causes, and treatment as “multifactorial” [4, 31], and identified care partners’ *behaviours* as one of the contributing factors [14]. We used the construct of “resilience as a process” to explore the association between care partners’ behaviours and OLWD’s fall risk [32]. This construct directed us to focus on the *process* through which care partners utilized and were impacted by internal (e.g., physical capacities, knowledge, self-efficacy) and external (e.g., financial support, social support) resources in managing fall risk for OLWD [33].

We drew from the theory of health behaviour in an ecological context to identify care partners’ behaviours relevant to FRM [34]. According to this theory, health promotion programs should focus on modifying health behaviours in a population at risk, as well as on 1. *health-related behaviours* taken by proximal others not purposefully that directly affect the population and 2. *health-protective behaviours* that are undertaken purposefully to foster the population’s health. This theory guided our exploration of two patterns of behavioural adoptions: behaviours adopted by care partners purposefully for managing fall risk, and behaviours care partners adopted without realizing their relevance to the FRM [34]. The distinction between behaviours adopted purposefully for FRM and those adopted without this expressed purpose might suggest the need for different behavioural change strategies for care partners. To assess the FRM behaviours that care partners adopted without FRM intention, a list of FRM behaviours was developed based on literature on care partners’ experiences of FRM for community-dwelling OLWD (See in Additional file 1) [15].

Guided by the literature and theoretical frameworks mentioned above, the research team developed an interview guide (Additional file 2) to collect data for creating a behavioural framework that conceptualized and organized the process through which care partners with different care responsibilities manage fall risk for OLWD.

### Sampling methods and recruitment

We used a sequential sampling strategy that was informed by the sampling approach of the grounded theory method [28, 35]. We began by selectively sampling to address inclusion criteria and then adopted theoretical sampling when concepts began to emerge [28]. Inclusion criteria were being an adult family member, friend, neighbour, or unpaid care partner who had primary or secondary care responsibilities for at least one community-dwelling OLWD in the prior two years. Criteria for defining a community-dwelling OLWD were 1. aged 55 years or older, 2. the care partner reported that a health care provider told them that the older adult had dementia, 3. score of 2 or higher on the AD-8 dementia screening tool [36] based on the care partner’s report, and 4. living in a private residence.

Community outreach methods [37] were used to recruit care partners from settings throughout Washington State, including outpatient clinics, community service organizations, and public health departments. Electronic and paper flyers were distributed via organizations’ mailing lists, public spaces, and staff. Potential participants contacted the lead researcher (Y.Z.) and underwent telephone screening to determine eligibility. The lead researcher was a Ph.D. candidate in social welfare and had 10 years of practice and research experience working with community-dwelling older adults and their families. In-person or remote interviews were scheduled for those who were eligible and wished to participate in the study. Of the 19 care partners who contacted the researcher, 14 completed the interviews after giving their informed consent to participate in the study. Two of the 19 dropped out after screening without reporting a reason; three of the 19 were paid caregivers (also not OLWD’s family members, friends, or neighbours) and thus ineligible.

Theoretical sampling is “the process of data collection for generating theory whereby the analyst jointly collects, codes, and analyses data” and “decides what data to collect next and where to find them, in order to develop a theory as it emerges” [38]. We communicated with community organizations about what types of care partners we sought to recruit based on our preliminary analysis of the first five interviews. As we recognized care partners’ experiences might differ based on their level of worry about OLWD’s fall risk and socioeconomic status, we intentionally recruited participants who did not perceive OLWD they cared for as “at high fall risk” and who were in low-income households. We also reached out to community organizations expressing the need to recruit secondary care partners. However, we still experienced difficulties recruiting care partners with secondary care responsibilities. Therefore, we were only able to conduct analysis on primary care partners’ reports about secondary care partners’ FRM behaviours that they might have observed.

### Data collection and analysis

Semi-structured interviews lasted an average of 90 (range: 30–240) minutes and were digitally recorded in care partners’ homes (*n* = 5), public spaces (*n* = 3), or by phone (*n* = 7). Two care partners had a second phone interview to answer follow-up questions. The OLWD was present during the interviews for three participants since care partners could not leave them alone. Participants received a $50 gift card. All the interviews were conducted from July 2019 to March 2020 by the first author (Y.Z.). Initial interview questions (Additional file 2) were informed by previous literature and relevant theoretical framework, focusing on 1. behaviours care partners adopted to manage OLWD’s fall risk, 2. behaviours they viewed as relevant to FRM, and 3. whether they had adopted any behaviours on the list (in Additional file 1). The list was developed based on the literature on dementia care partners’ experiences of FRM [15] and administrated at the end of the interview to capture any FRM behaviours that care partners adopted without realizing the relevance to OLWD’s fall risk. The interviewer took field notes during and after each interview, recording the interviewer’s observation of the interview interactions, any interesting information that caught the interviewer’s attention, and the interviewer’s thinking process [26].

To keep researchers’ eyes open to all kinds of observation and aspects [26], all participants were given multiple opportunities to express a range of behaviours that they adopted or observed. They were asked open-ended questions about their behaviours and presented with the list of behaviours derived from the literature and asked what might be missing. A total of four types of new behaviours were identified through conducting preliminary analysis of interviews and field notes with the first five participants: “receive help from other care partners”, “mobility assistance”, “assess and address the OLWD’s health conditions”, and “supporting OLWD’s help-seeking behaviours”. As each interview identified one of these new behaviours, it was immediately added to the list for subsequent interviews. After the fifth interview, no new behaviours emerged during the preliminary analysis. This process meant that we might have undercounted the number of participants who adopted or observed these specific behaviours because the first five participants did not see them all listed on the interview prompt list. However, this method of allowing the initial interviews to inform the interview guide aligned with methods of the grounded theory approach because it acknowledged that researchers could not have a fully complete a priori knowledge of the phenomenon [26, 28].

After completing data collection, the first author used coding procedures based on grounded theory methods [28] to conduct a systematic analysis on transcribed interviews using Atlas.ti 9.0.5 to develop a more nuanced understanding of different care partner behaviours relevant to FRM. First, we used the line-by-line initial coding and memos [28] to begin to identify FRM experiences, considerations, procedures, and reflections by care partner respondents. This resulted in 234 open coding categories (for example, “advocate for OLWD’s needs with other care partners and care providers”, “assess OLWD's health condition after fall to make care decisions”, “be mindful about everyday activity arrangement”).

Following the initial coding, the focused coding was conducted to refine and tentatively categorize initial codes that indicate analytic significance [28]. We identified categories of these behaviours based on 1) the meaning of these behaviours, 2) how these behaviours were attributed to the participant (primary care partners) or other care partners (secondary care partners), 3) how these behaviours were adopted purposefully or not purposefully for FRM, and 4) how these behaviours were adopted at different temporal stages of fall risk management. Both frequent codes and infrequent codes of FRM behaviours were included to develop a comprehensive understanding of care partner behaviours.

For the third step, the axial coding was conducted to specify the properties and dimensions of each category [28]. This analysis procedure resulted in a preliminary behavioural framework that described eight domains of FRM behaviours, purposefulness, different behavioural patterns of primary and secondary care partners, and four stages of the FRM.

Lastly, using the constant comparative process, the first author compared the preliminary behavioural framework with each interview, memo, and relevant literature to develop definitions for each behaviour category [26, 28]. To understand patterns of behaviours and how common each was relative to others, the number of primary care partners who adopted each behaviour purposefully or not purposefully was summarized in a matrix. FRM behaviours adopted by other care partners based on the participant’s observation were identified; however, intentionality was not categorized given that their intention was not able to be determined. We have also identified four stages of FRM and mapped which behaviours were adopted at each stage of FRM. Our multidisciplinary research team formed by scholars in social work, nursing, medicine, and occupational therapy reviewed and refined the framework and definitions.

The first author completed the data analysis in consultation with members of the study team (C. B. & T. S.) who had expertise in conducting qualitative studies with older adults and their caregivers. To reduce the potential for bias, we adopted extra steps to ensure the rigour and trustworthiness of the analysis. First, the full research team reviewed and approved the data collection protocol and analysis procedures, and contributed to the interpretation of findings. The constant comparison process was employed by returning to the data three times to develop and verify categories of this behavioural framework during the analysis. Additionally, all interviews were coded beyond the point of conceptual saturation to reduce the potential for coder bias and to ensure that all possible FRM behaviours were captured and categorized. The first author also wrote methodological and theoretical memos to update the interview guide and develop analysis results throughout the data collection and analysis period. Raw data (recording, transcripts, field notes), coding schema, coded transcripts, summary products, data analysis meeting notes, and theme reports were filed by date to provide an audit trail.

## Results

### Participants

Care partners lived across four counties in Washington State (San Juan, King, Snohomish, and Spokane). Characteristics of participants and OLWD were shown in Table 1. All 14 participants interviewed were care partners in the primary caring role. Nine of them reported FRM behaviours of secondary care partners, including 16 family care partners and some neighbours and friends.

**Table 1:** Characteristics of participants

		**Characteristics of care partners (CP)**							**Characteristics of older adults living with dementia (OLWD)**				
**CP’s Pseudo name**	**Interview format**	**Age**	**Gender**	**Race/ethnicity**	**Education**	**Employ-ment**	**Relationship to OLWD**	**OLWD’s other CP**	**Age**	**Gender**	**Dementia type**	**Dementia stage**	**Fall history in the past**
Tracy	Phone	75	Woman	Non-Hispanic white	Some college	No	Adult child	NR (Not reported)	95	Woman	Unspecified	Mild	Yes
Leila	In-person (public space)	80	Woman	Non-Hispanic white	Completed college	No	Spouse /partner	Two sons	79	Man	Parkinson's Dementia	Mild	Yes
Emma	In-person (CP’s home)	66	Woman	Non-Hispanic white	Completed college	No	Spouse /partner	One daughter and one son-in-law	69	Man	Alzheimer’s Disease	Moderate	No
Betty	In-person (CP’s home)	73	Woman	Non-Hispanic white	Completed college	Part time	Spouse /partner	One son	75	Man	Parkinson's Dementia	Mild	Yes
Marshall	In-person (public space)	71	Man	Hispanic	Some college	No	Spouse /partner	One daughter	73	Woman	Alzheimer’s Disease	CP did not know	No
Marissa	In-person (CP’s home)	60	Woman	Non-Hispanic white	Some college	Part time	Adult child	One daughter	92	Woman	Alzheimer’s Disease	Severe	Yes
Monica	In-person (CP’s home)	58	Woman	Non-Hispanic white	Completed college	No	Adult child	One son-in-law, and some neighbours and friends	94	Woman	Unspecified	Mild	Yes
Teresa	Phone	48	Woman	Non-Hispanic white	Completed college	No	Adult child	One daughter and one son	81	Woman	Alzheimer’s Disease	Moderate	Yes
Shannon	In-person (CP’s home)	66	Woman	Black	Completed college	Part time	Adult child	One grandson and one grand-daughter	85	Woman	Vascular dementia	CP did not know	Yes
James	Phone	87	Man	Not reported	Completed college	No	Spouse /partner	One daughter and one granddaughter	83	Woman	Dementia with Lewy Body	Moderate	Yes
Jane	Phone	73	Woman	Non-Hispanic white	Some college	No	Spouse/ partner	NR	76	Man	Alzheimer’s Disease	Moderate	Yes
Veronica	Phone	60	Woman	Asian and non-Hispanic white	Some college	Full time	Adult child	NR	83	Man	Alzheimer’s Disease	CP did not know	Yes
Glenn	In-person (public space) /Phone	85	Man	Non-Hispanic white	Completed college	Full time	Spouse/ partner	One son, one daughter, and some neighbours	83	Woman	Frontotem-poral dementia	Moderate	Yes
Catherine	Phone	64	Woman	Non-Hispanic white	Completed college	Full time	Adult child	NR	86	Woman	Alzheimer’s Disease	Moderate	Yes

### A behavioural framework for care partners’ FRM

**Fig. 1:**
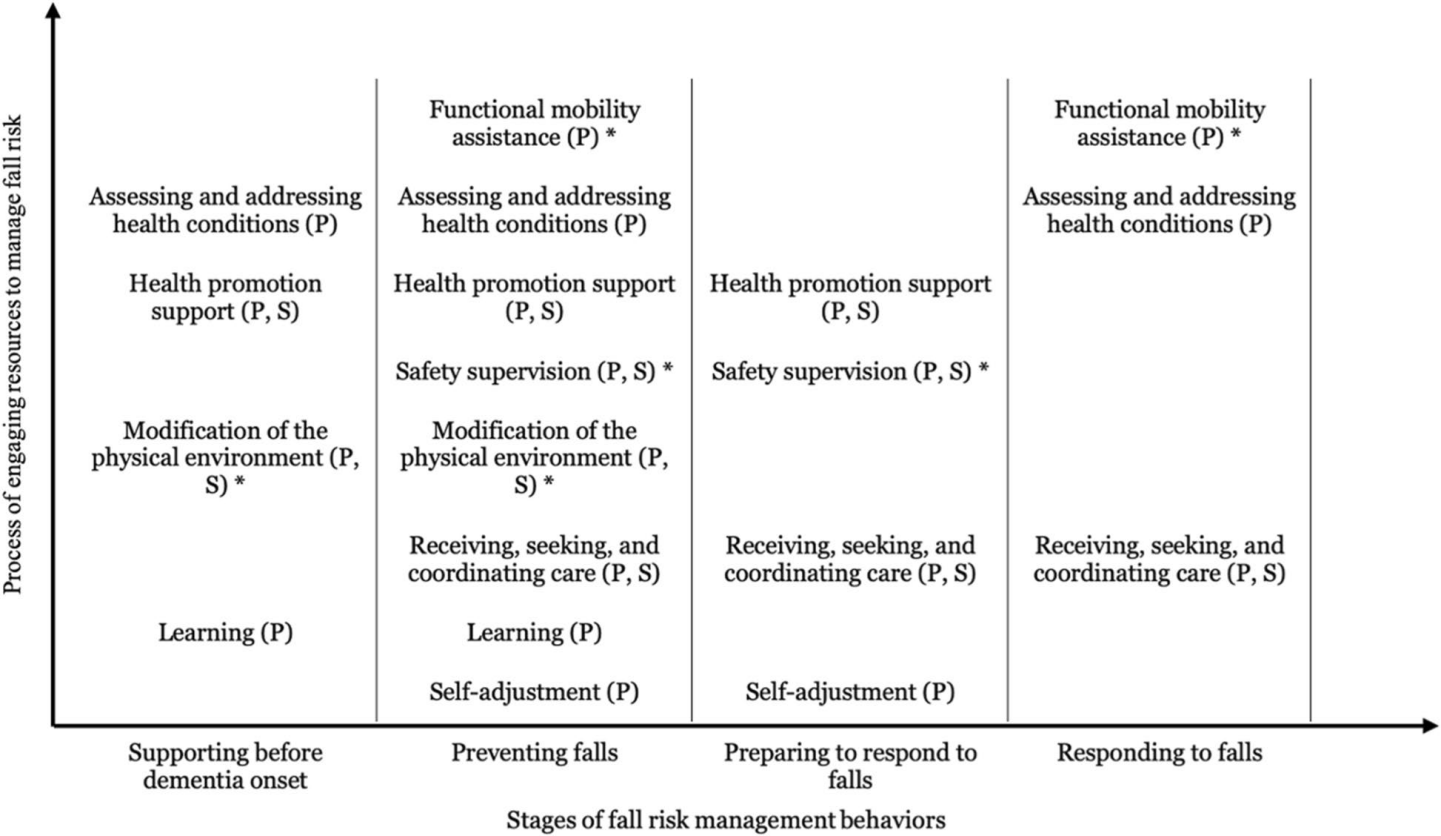
A behavioural framework for dementia care partners’ fall risk management. *Note.* P: behaviour adopted by primary care partners; S: behaviour adopted by secondary care partners; *: behaviour most often adopted purposefully by primary care partners for fall risk management (others were adopted but not expressly for fall risk management)

Two dimensions of a new behavioural framework emerged (Fig. 1). The first dimension was the process of engaging resources to manage fall risk. This process consisted of eight distinct behavioural domains and a total of 36 FRM behaviours. The second dimension consisted of four temporally distinct stages related to fall prevention and actual fall occurrence.

### Process of engaging resources to manage fall risk

The eight behavioural domains of FRM, their definitions, and associated behaviours were presented in Table 2 along with the frequencies of the behaviours. Primary care partners engaged in behaviours from all eight behavioural domains of FRM. According to primary care partners’ reports, secondary care partners engaged in behaviours from several of the eight domains, including health promotion support, safety supervision, modification of the physical environment, and receiving, seeking, and coordinating care. Primary care partners often provided functional mobility assistance, safety supervision, and modification of the physical environment purposefully for managing fall risk. They also adopted other FRM behaviours without realizing their relevance to FRM. These behaviours included assessing and addressing health conditions, health promotion support (especially enhancing activity engagement, exercise support, and diet support), and receiving, seeking, and coordinating care. Table 2 included additional exemplary excerpts from interviews for each of the 36 behaviours.

**Table 2:** Eight domains of dementia care partners’ fall risk management behaviours

Domain	Definition	Behaviour	Frequency ^a^			Exemplary excerpt
			**Primary care partners (CP)**		**Secondary CP**	
			**Purposefully**	**Not purposefully**		
**Functional mobility assistance**	Physically or verbally assist older adults with dementia to move around in the environment in order to participate in the activities of daily living safely [39]	Standing assistance	6	1	0	“She has a hard time operating her foot then she will hang on to something or she will hang on to me. But no cane. (Tracy)” “(After my wife fell), I first make sure she was all right and got her back to her feet.” (James)
		Walking assistance	7	1	0	“I would just put my hand on her arm. I would just hold on to her a lot when we are walking outside.” (Teresa)
		Toileting assistance	2	0	0	“It was riskier as she tried to lean on the toilet and fall off. I’ve come to the door and ask her: Do you need my help? She could make the attempt and I took over when she needed it.” (Marissa)
		Shower/bath assistance	1	1	0	“It was really hard to get her into the bathtub without her falling or I falling. I had to have a strategy planned around how my body would work to help her body work.” (Marissa)
		Hazard reminder	4	0	0	“When he’s walking up to church or walking up to the doctor’s, I would say, ‘there’s the curb, be careful, pick your feet up.’” (Jane)
		Mobility restraint	3	0	0	“If she goes walking out for any distance, I wouldn't allow her to do that by herself.” (Tracy)
**Assessing and addressing health conditions**	Assess and address older adults with dementia’s mental and physical health conditions directly	Assess physical and mental health conditions	12	7	0	“I look at my mom, and I see she's getting weaker. As she walks, she's not picking up her feet. She just doesn't have much strength or energy.” (Catherine)
		Address physical and mental health conditions	5	3	0	“I think that fall was because of the UTI.…. The urologist gave us antibiotics. But then we went back in 10 days, and she still had it and so then she gave mama more antibiotics and that cleaned it up.” (Monica)
		Medication management	2	10	2	“I had to figure it (the side effect of medication) out by watching her reactions…. there was something about it that seemed to make her a little loopier. I just told the doctor either you prescribe her something else, or I'll just take her off of it.” (Marissa)
**Health promotion support**	Care partners enable older adults living with dementia to increase control and improve their health [40]	Health and risk communication	12	1	0	“I just told her I said we don't get any exercise. Let's go get some exercise. I said we both had diabetes. You know, it will help your Alzheimer's. It will help with our diabetes. I said, look, let's go do it (a balance exercise program). And she said, she thought about it. Next day, she says I'm ready. Let's go.” (Marshall)
		Enhance activity engagement	5	7	5	“Our sons will come and stay with him. They do a lot of playing. They like to play poker together. (Leila)” “We go up and down the stairs so that she can get the dogs food. She has to feed the dog. That's her job. I gave her that job. I hope it gives her a sense of responsibility.” (Marshall)
		Exercise support	3	7	4	“I just give her instructions as to how to do them if it’s a new one. If it’s a new exercise, I show her how to do it.” (James)
		Diet support	2	14	5	“I try to keep her nutrition. It is not likely to stop her eating things that you think is not nutritious, but you can provide you more food with more nutrition.” (Tracy)
		Improve and maintain self-efficacy	3	2	1	“She used to work around the house, and you know, the yard and she feels like she's useless now. And I said, no, you're far from useless because you have a full-time job. That is not to fall.” (Monica)
		Improve help-seeking behaviours	2	1	0	“I told my wife: knowing what your limits are is extremely important. Don’t be macho about oh, I can still do that or whatever. And don’t be embarrassed to ask others to help you.” (Glenn)
**Safety supervision**	Being able, ready, and willing to perform intentional acts to reduce injury risk to a less capable person [41]	Pay close attention	8	1	0	“When we're walking, he walks really good, but I just have to make sure I keep him in sight.” (Emma)
		Regularly check in	2	0	2	“(When I left the house), I often call her to check on her. She could fall and nobody be able to be here for the doors locked and just not eating properly.” (Tracy)
		Be present	5	1	5	“Well, I think the most important thing that I did all around to manage fall risk for my mom was to be there for her, make her happy and make her feel safe.” (Marissa)
**Modification of the physical environment**	Modify the physical aspect of the environment that OLWD interact with	Home safety assessment, modification, and organizing	12	2	4	“I just keep things picked up. I would not want to see papers on the floor or anything like that. That would be a trip hazard. And sometimes when he likely finishes the newspaper, he drops it on the floor. I picked it up so that it won't be a danger.” (Leila)
		Ensure footwear safety	2	0	0	“(My husband) has fallen when he was hiking, and the soles came off of this shoe…. We had to have all their soles replaced.” (Emma)
		Support use of mobility assistive devices	6	2	3	“I know there's a risk of falling so I do encourage him to use his walker.” (Leila)
		Support use of monitoring technology	4	0	0	“I also have a medical alert on her so that if I don’t happen to be right where she is at the time, she will get medical attention immediately.” (James)
**Receiving, seeking, and coordinating care**	Receive, seek, and coordinate formal and informal care and services	Use of informal care	6	4	4	“Like on Saturday, I went to the conference the whole day. I left at 8:30, and our neighbour came over at 10. One of my girlfriends came over at 11:30; and my other girlfriends came over at 1:00. And then our neighbour came back over at 2:30. For every hour, someone came over to stay with my mom.” (Monica)
		Hire and coordinate with paid caregivers	0	2	0	“I have a caregiver that comes on Mondays and Wednesdays for from about 9:30 until 1:00, okay, he takes him out for a walk… and he helps with his toilet.” (Emma)
		Use of social and health services	8	13	2	“These things got scary for her to be walking down the street. I got her a new assessment that gave her hours in the adult day care centre. I could pick her up after I am done working.” (Shannon)
		Communicate with other care partners and professionals	4	0	1	“I finally got my mom convinced to remove the rugs that, 'You have to compromise, mom (the secondary care partner). You can’t just have the house like you want it. We have to make it safe. Not just for dad (OLWD), but for you too.'” (Veronica)
**Learning**	Gain information, knowledge, and skills from past experience and the outside world about fall risk management for OLWD [32]	Learn from professional care providers	8	6	1	“We had an occupational therapist and a physical therapist come here to the house. …They taught me exercises to do with her, because they would come like once or twice a week.” (Monica)
		Learn from care partners’ life experiences	8	4	0	“With my car accident, when I finally graduated from the wheelchair, I was falling. Every single day, probably 15 times a day. I had to learn how to mitigate. That’s how I learned how to fall so I taught my mom how to do that when she fell.” (Marissa)
		Learn from older adults living with dementia	6	2	0	“We were walking along, and I said, oh, I wanted to tell you about the program on TV tonight. He said, wait a minute, I can't do two things at once. So, I learned to be careful if he's concentrating on something and not to try to interrupt him.” (Leila)
		Learn from social network	6	5	0	“The biggest thing for the support group is talking to people who have had gone through it with their parents. They tell me different strategies that they use to help prevent falls.” (Marissa)
		Learn online and media	3	1	0	“I started to do research online of all the different providers of these fall alerts, trying to figure out, who does have reliable phone service when somebody falls in this particular location.” (Tracy)
**Self-adjustment**	Care partners change their arrangements of living, sleeping, working, and social and physical activities in response to the fall risk of older adults with dementia	Change living arrangement	0	4	0	“That's scary, if there was a problem, she couldn't call 911. If she couldn't figure out the phone, we realized that it was not safe for her to be alone all the time. So, we moved her in with me.” (Teresa)
		Change sleeping arrangement	1	0	0	“She just lost her balance. So I thought, okay, I better come in the house. So I slept on her bedroom floor.” (Monica)
		Reduce working time	1	1	0	“For the recent cancer diagnosis, he was so distracted; something took over his mind, so he might not pay much attention to his body… I stopped working because my husband needed me here 24 seven.” (Betty)
		Avoid social activities	0	5	0	“I had nobody on Sunday before. I used to be very active in my church. Now I cannot go to church.” (Shannon)
		Adapt walking behaviours	4	0	0	“I remember in my mind; you don't want to say hurry up. I don't want her to feel anxious. If she does, she's gonna fall.” (Marshall)

^a^These numbers represents how many care partners reported that they adopted each behaviour or reported that that secondary care partners did

#### Functional mobility assistance

Primary care partners reported physically or verbally assisting OLWD with movements to perform activities of daily living safely (e.g., assistance to stand, walk, toilet, and shower/bath) [39, 42]. As one participant explained: “I noticed my mom tried to lean forward on the toilet and fall off. …I would come to the door and ask her if she needs my help…. I would let her do whatever she could do on her own but took over when she was about to fall.” (Marissa).

Care partners described how their OLWD’s cognitive and visuospatial impairment impacted how older adults perceived, analysed, and interacted with the physical environment, especially the outdoor environment. Therefore, care partners needed to provide reminders about hazards, especially when outside, even if the OLWD still had good physical functions. One participant said, “When we were out, I just tried to, particularly on the sidewalk, stay right beside him. I would say, oh, there is an irregular place here, you know, just some verbal cues to him.” (Leila).

When care partners could not provide such assistance, they reported restraining their OLWD from going out or doing activities alone. One care partner asked OLWD to not go out when she was at work:

*What worried me the most was his falling. I worried about that all the time because he still died thinking that he could do all this stuff. I mean, he went hunting by himself. I said, “Dad, don’t go out by yourself. Wait until I’m home.” …And he’d be like, “Yes, mom. Yes, mom.” I’m like, “Dad, I’m not trying to ‘mom’ you. I just worry. I worry about you falling and now I don’t know where you are.” (Veronica)*

#### Assessing and addressing health conditions

Primary and secondary care partners engaged in assessing and addressing OLWD’s mental and physical health conditions relevant to fall risk. One participant shared that she noticed some symptoms her husband had that raised her concern about his risk of falling: “He just forgot the mechanics of how to move his body. I could see this would make him fall in the future.” (Emma) Additional conditions noted by care partners included visual impairment, arthritis, gait changes, mobility impairment, fatigue, depression, spatial awareness difficulty, dual-task attention, cancer, urinary tract infections, risk of heart failure, diabetes, behavioural and psychological symptoms of dementia (BPSD), and fall injuries.

To address these health conditions, care partners engaged in medication management and a variety of non-pharmacological approaches, such as psychosocial support, art, gardening, and outdoor recreational activities in nature. One daughter (Shannon) shared how she used music and a lavender diffuser when the OLWD was anxious and resistant to walking, eating, sleeping, or taking medications. Despite the prevalence of these behaviours, only two primary care partners reported managing medication and using non-pharmacological approaches to purposefully address OLWD’s fall risk.

#### Health promotion support

In addition to assessing and addressing OLWD’s health issues, both primary and secondary care partners tried to enable OLWD to increase control and improve their health [40]. Primary care partners shared their roles in discussing health and safety concerns with OLWD, enhancing activity engagement, providing exercise and support, improving and maintaining OLWD’s self-efficacy, and supporting their help-seeking behaviours. Secondary care partners often supported OLWD’s activity engagement, exercise, and diet.

Primary care partners played an important role in communicating health and risk issues with OLWD to support them in adjusting their behaviours and making decisions. They provided instructions to adjust OLWD’s walking habits, taught them how to fall and how to get up after falling, discussed potential ways to address fall risk, and modelled desired behaviours. One wife care partner shared how she had a conversation with her spouse after he had multiple falls: “It was maybe the second or third time where I said, okay, you need to be more conscious of this. How can you figure out a way so that you don't fall? He looked into this and then came up with the strategy: stop when you are shuffling, and you take time to think then walk normally.” (Betty).

Primary care partners often provided exercise and diet support without realizing the associations between these behaviours and OLWD’s fall risk reduction. However, care partners described enhancing OLWD’s activity engagement, which was defined as care partners supporting OLWD’s participation, spending time, and gaining positive affective experiences in everyday activities [43], as relevant to FRM. Sometimes care partners had to adapt these activities to the capacities of OLWD. One participant shared: “One day, my mom suddenly started pulling everything out of the freezer. I know the freezer didn’t need to be cleaned out. But who cares? We wiped it down. And then she put everything back in. We had a super fun afternoon.” (Marissa) The participant further explained how these creative activities made her mom feel that her limitations were accepted; therefore, her mom seemed comfortable asking the participant for help in risky situations.

Care partners also reported the importance of doing activities that could improve and maintain OLWD’s self-efficacy, which might reduce the fear of falling. One care partner explained why he believed elevating OLWD’s self-efficacy helped with fall risk reduction: “(Going to her favourite restaurants) makes her feel good about herself. And I think that helps. I think it makes her mind say I am. I'm good. I'm okay. We all need a boost of confidence from time to time. You want someone to tell you, you're doing a good job.” (Marshall).

Care partners described efforts to increase OLWD’s help-seeking behaviours. For example, one participant shared how she came up with ways to encourage her dad to ask for help:

*My dad can fall and stay on the ground for two hours, even though he got the fall alert. He thinks I can do this myself. This is the hardest part for him even though I kept asking him, why don't you call us for help? He always thought I didn’t want to be a burden. I saw a thing, so I printed it out to put in dad's bathroom –“You are not a burden. You are carrying a burden, which, by definition, you cannot do alone.”* (Veronica)

These examples of care partners’ behaviours in supporting OLWD’s health promotion demonstrated that they not only offered direct assistance but also tried to support OLWD’s health-promoting self-care and help-seeking behaviours.

#### Safety supervision

Primary care partners took extensive efforts in safety supervision for OLWD, defined as “being able, ready, and willing to perform intentional acts, such as restraint, guidance, modelling, or instruction, as needed to reduce injury risk to a less capable person [41].” Care partners’ safety supervision included three distinct behaviours: paying close attention, regularly checking in, and being present. Secondary care partners also helped with regular check-in and staying present with OLWD, especially when primary care partners were not available.

Paying close attention referred to care partners’ efforts to keep OLWD in sight constantly, especially when older adults were moving and doing anything that might have a high fall risk, such as taking a bath or carrying things. Care partners often used this strategy when OLWD did not accept assistance:

*I tried not to do it too much because she (my mom) still likes being independent. And that would irritate her if I constantly helped her with everything. I'd make sure that if she were walking from the bathroom to the front door or to the kitchen, I would strategically sit here when she is wobbly.* (Marissa)

When care partners had to leave OLWD alone, care partners regularly checked in to ensure OLWD’s needs were met and to determine whether they were safe to avoid OLWD’s long lies on the ground following a fall. Some care partners believed that just being present with the OLWD could help prevent falls. A participant shared: “I think the most important thing that I did all around to manage fall risk for my mom was to be there for her, make her happy and make her feel safe.… When I am around, you can see that peace on her face.” (Marissa).

#### Modification of the physical environment

Both primary and secondary care partners made various efforts to modify the physical aspects of the home, ensure footwear safety, and encourage the use of mobility assistive devices and monitoring technology. This involved not only large-scale modifications of stairs, rugs, and toilet seats but also ongoing organizing to reduce hazards (e.g., picking up papers on the ground). Care partners gathered the information for mobility devices (e.g., walker, cane, wheelchair) and monitoring technology (e.g., Global Positioning System tracking via cell phone, personal emergency response systems, door sensors). They made decisions about which type of device or technology to obtain, purchase or install. Care partners also attempted to get OLWD to use these devices or tools. For example, one participant shared how she persuaded her mom to feel comfortable using a walker and constantly reminded her mom to use it: “I tell her that she needs to look at it as a tool and she's still the boss. That's why we call it the wheels. We don’t call it a walker…. I just need to make sure she uses her wheels. Sometimes, she forgets because she is so independent. I have to remind her.” (Monica).

#### Receiving, seeking, and coordinating care

Primary care partners described receiving, seeking, and coordinating different care and services, including informal care, paid caregiving, and social and health services, for managing OLWD’s fall risk. Care partners often asked for and received help from other families or friends, especially when they were distant. They also discussed OLWD’s health conditions and prepared for emergency needs with other care partners. Care partners hired and coordinated with paid caregivers to provide company to older adults, assist with household organizing, and enhance OLWD’s daily activities. They used social and health services for OLWD to address fall risk (including health conditions related to fall risk) or fall events. These included taking older adults to community exercise or nutrition programs, accompanying older adults to doctor’s appointments, selecting physical therapists for older adults, purchasing personal trainers services, looking for health services after older adults fell, and accessing hospice services.

Care partners also tried to communicate with other care partners and service providers about the need to address OLWD’s risk of falling. For example, one participant shared how she was able to advocate for her mom when using services from an adult day care centre:

*When they bring my mom off the day-care centre bus, I expect them to assist her because she needs help. It’s dark, but they didn’t walk my mom up to the door. I think there is a risk of falling if she doesn’t have assistance. So, I communicated with the staff on the bus…. I also asked them to provide occupational therapy services to her as they promised.* (Shannon)

In this way, care partners used their knowledge, intuition, and experiences to challenge OLWD’s social environment and service provision status quo.

#### Learning

Primary care partners emphasized the importance of their learning, a cognitive-behavioural process in which they gained information, knowledge, and skills to address the needs of OLWD and themselves [32]. Some care partners learned how to manage fall risk from professional care providers, such as learning how to engage OLWD in exercises from occupational therapists and physical therapists. They learned about fall risk from their own life experiences, such as caring for children and other family members, and even their own experiences of falling and living with disabilities. One participant described how his experiences of disabilities allow him to know how to walk with OLWD: “I had an accident many years ago when I was a construction worker. Something fell and hit my face. For a long time, I was living with a disability. I know how frustrated I was. I don’t want to rush her (OLWD) when she walked very slowly. …She is living with a disability that people cannot see.” (Marshall).

Care partners also reported how they learned about managing falls from OLWD. One participant explained she learned from her mom a safer way to walk and then used this knowledge to continue to support her mom as dementia progressed: “My mom is the one that came up with ‘bending their knees’ after she saw a man and his wife walking a long time ago and they were both walking with their knees bent. I just need to keep reminding her of that.” (Monica). Social networks, such as caregiver support groups, were another essential resource for care partners to learn about FRM. One participant also shared how he learned about FRM and other health management skills from TV shows and the internet. Care partners accessed a wide array of resources to develop their capacity to manage fall risk for OLWD.

#### Self-adjustment

Primary care partners reported that they needed to adjust their living and sleeping arrangements, work, and social and physical activities to manage OLWD’s fall risk. Care partners said they could no longer able to work full-time, meet with friends, go to dancing classes, or participate in community events when they noticed that OLWD experienced a high risk of falling. Self-adjustment also included care partners changing their walking habits (such as chatting when walking) and walking speed when walking by the OLWD.

### Stages of FRM

This study revealed four stages of FRM: 1. supporting before dementia onset, 2. preventing falls, 3. preparing to respond to falls, and 4. responding to falls. Each FRM behaviour might be adopted for challenges specific to different stages of FRM, as depicted in Table 3.

**Table 3:** Behavioural domains and behaviours according to stages of fall risk management

Domain	Behaviour	Stage of fall risk management			
		**Supporting before dementia onset**	**Preventing falls**	**Preparing to respond to falls**	**Responding to falls**
**Functional mobility assistance**	Standing assistance		√		√
	Walking assistance		√		√
	Toileting assistance		√		
	Shower/bath assistance		√		
	Hazard reminder		√		
	Mobility restraint		√		
**Assessing and addressing health conditions**	Assess physical and mental health conditions	√	√		√
	Address physical and mental health conditions		√		√
	Medication management		√		
**Health promotion support**	Health and risk communication		√	√	
	Enhance activity engagement		√		
	Exercise support	√	√		
	Diet support	√	√		
	Improve and maintain self-efficacy		√		
	Improve help-seeking behaviours		√	√	
**Safety supervision**	Pay close attention		√	√	
	Regularly check in		√	√	
	Be present		√	√	
**Modification of the physical environment**	Home safety assessment, modification, and organizing	√	√		
	Ensure footwear safety		√		
	Support use of mobility assistive devices	√	√		
	Support use of monitoring technology			√	
**Receiving, seeking, and coordinating care**	Use of informal care		√	√	√
	Hire and coordinate with paid caregivers		√		
	Use of social and health services		√		√
	Communicate with other care partners and professionals		√		
**Learning**	Learn from professional care providers	√	√		
	Learn from care partners’ life experiences	√	√		
	Learn from older adults with dementia		√		
	Learn from social network	√	√		
	Learn online and media	√	√		
**Self-adjustment**	Change living arrangement		√	√	
	Change sleeping arrangement		√	√	
	Reduce working time		√		
	Avoid social activities		√		
	Adapt walking behaviours		√	√	

#### Supporting before dementia onset

Some care partners started to engage in the following domains of FRM behaviours before cognitive impairment had progressed to the OLWD needing help. These efforts included assessing physical and mental health conditions, exercise and diet support, home safety assessment, modification and organizing, support use of mobility assistive devise, and learning from different resources. For example, one participant helped modify the home environment when her mom had a fall-related fracture before experiencing cognitive impairment. Others emphasized their roles in encouraging exercise and a healthy diet for OLWD as they grew older together.

#### Preventing falls

All the identified FRM behaviours were found to be relevant for this stage.

#### Preparing to respond to falls

Care partners began to prepare to respond rapidly to fall incidents when they sensed that the chances of falls were increasing. One participant described “(The OLWD) will fall no matter what I do—it is just a matter of time. I don’t know how you can prevent it.” (Jane). In this stage, care partners focused on health and risk communication, improving older adults’ help-seeking behaviours, adopting behaviours that enhance safety supervision, using monitoring technology, and seeking informal support. They also started to conduct more self-adjustment, such as reducing their work time and social or recreational activities to be available to respond promptly when falls occurred.

#### Responding to Falls

Participants described taking immediate actions to assist OLWD when falls occurred. They helped OLWD in standing and walking (if able), assessing and addressing any injuries and making decisions about seeking medical attention.

## Discussion

There is little evidence to suggest how dementia care partners can most effectively engage in FRM. This study was the first step towards addressing this essential but understudied issue by proposing a multi-domain, multi-stage behavioural framework of FRM for primary and secondary care partners of community-dwelling OLWD. We have identified 36 behaviours that fit into eight distinct, empirically derived domains. Four stages of FRM were identified to describe the trajectory of care partners’ FRM. Primary and secondary care partners both participated in FRM but took on different roles, and different FRM behaviours might be adopted by care partners with different intentions.

### Eight domains of FRM behaviours

This study identified and categorized care partners’ FRM behaviours that had not been described or well-characterized previously, especially those understudied behaviours of functional mobility assistance, assessing and addressing health conditions, learning, and communicating with other care partners and health professionals.

Previous studies suggested that OLWD without functional mobility assistance were at higher risk of nursing home placement and mortality [44]. Our findings explained how care partners provided mobility and daily living assistance to reduce these risks. As cognitive and visuospatial impairment is associated with a high risk of falling for OLWD, OLWD curtail going outside due to anxiety and fear when they still have good physical functions [45]. Therefore, care partners’ engagement in hazard reminders may be critical to ensure OLWD’s ongoing engagement in outdoor activities, contributing to the fall risk reduction and delayed adverse consequences of falls [46]. As functional mobility assistance is often undervalued and described as mundane care works [47], care partners received little training in providing this type of assistance [48, 49]. This study indicates that future FRM interventions should incorporate support for care partners in providing functional mobility assistance to OLWD.

Fall risk among OLWD is often associated with various physical and mental health conditions [4], requiring medical and non-pharmacological interventions [47, 50, 51]. Previous research suggested that care partners had limited insights into the risk and protective factors related to falls for OLWD [15]. However, in this study, some care partners noted that OLWD might be at risk of falls by observing their gait, balance, urinary tract infections, dual-task attention, and concerning behaviours early on during their caring process. One study suggests that the unmet need for falls/rehabilitation assessment for OLWD is a common cause of falls that leads to adverse events [52]. Given the absence of validated tools and specific recommendations to assess fall risk for OLWD [4, 53], this finding revealed opportunities to engage care partners to develop early fall risk detection and assessment for community-dwelling OLWD and to provide training and supervision for care partners to address modifiable predictors of falls in OLWD.

There is currently limited evidence about specific successful strategies for enhancing care partners’ learning in managing fall risk for OLWD [32]. Our study indicated that future FRM interventions should not view care partners as “empty vessels'' [54] but rather facilitate care partners’ learning by integrating their life experiences, social networks, internet and media, and interactions with different social and healthcare providers. Furthermore, in some instances, care partners learned directly from OLWD about what measures could reduce their risk of falling, especially at the early stage of dementia progress. This finding suggested the importance of initiating communication and enhancing the mutual learning between older adults and their care partners about FRM at the early stage of the dementia diagnosis.

In addition to learning from others, we also found that care partners made distinctive and proactive contributions to improving the social environment and social and health services for OLWD based on their lived experiences of where the gaps were, as was stated in a previous study [55]. This finding suggested that strengthening the communication among care partners and service providers might not only directly benefit OLWD and their care partners. It might also help service providers to identify institutional factors that are associated with a high risk of falling for OLWD [56] and strengthen institutional capacity in fall prevention at home and community-based service settings [57].

### Distinctive roles of primary and secondary care partners in FRM

Although having two or more care partners for OLWD is a common phenomenon [19], few studies have examined the similarities and differences between primary and secondary care partners in FRM behaviours. Previous studies revealed that multiple care partners who provided support for the same OLWD often encountered challenges in distributing care responsibilities, receiving consistent guidance, and collaborating on care tasks [19, 58]. We found that secondary care partners also played a facilitatory role in FRM, including supporting OLWD’s needs in health promotion, safety supervision, modifications of the physical environment, and accessing social and health care. Fall prevention programs should incorporate strategies, such as family conferences [59] or digital communication tools [60], to support such collaborations across the care network of OLWD.

### Engage FRM behaviours adopted with different intentions

Purposefully adopted behaviours for FRM might be related to care partners’ perceptions and knowledge of fall risk among OLWD. However, it is unclear what factors and mechanisms might shape FRM behaviours not purposefully adopted by care partners, which might require different intervention strategies. Further exploration is needed to understand how to engage care partners to adopt FRM behaviours adopted with different intentions, even if they do not have extensive knowledge and/or strong motivation in FRM. This finding demonstrated potential opportunities to develop FRM interventions that built upon and reinforced behaviours that care partners might engage in for reasons not specific to the fall risk. For example, as we found that many care partners engage in medication management without realizing that it was relevant to FRM, future fall prevention programs should fill in the gap by assisting care partners in addressing the fall risk that is related to OLWD’s medication use [61].

### The trajectory of care partners’ FRM

Finally, this behaviour framework highlighted the trajectory of care partners’ FRM for OLWD, especially the early engagement of care partners in health management for older adults even before dementia was apparent. The framework we described herein suggested that care partner FRM behaviours included both preventing falls and responding to fall events. Care partners’ behaviours might help mitigate the severity of fall-induced injuries [62]. Care partners cued OLWD about safe landing strategies and help-seeking after falls, which were techniques rarely incorporated into FRM interventions for OLWD [63]. Other studies also found that care partners provided immediate assistance when OLWD fell, despite no prior training to do so without causing injuries to OLWD or themselves [64]. The lack of training for care partners might cause injuries among family care partners, which received little attention [65].

Care partners often adopted self-adjustment strategies when they felt the need to put more effort into detecting falls and preparing to respond to older adults’ fall incidents rapidly. However, these self-adjustments, especially reducing working hours, self-care activities, and social activities might negatively impact care partners’ health and well-being [66–68].

Previous studies offered divergent perspectives on how to respond to care partners’ belief that a fall was inevitable. Some studies suggested the need to modify such perception since it might thwart care partners’ motivation to manage fall risk for OLWD [54]. Another study suggested that professional care providers needed to understand how care partners’ risk management behaviours were sometimes based on fatalism and unpredictability to collaborate effectively with care partners [69]. Recently, there were increased discussions about whether and when it would be appropriate to shift focus from fall prevention efforts to a more palliative approach [4]. We found that although some care partners did hold the belief that OLWD’s falls were not avoidable, they still needed support in FRM related to learning how to protect themselves when assisting OLWD to get up from the floor, assessing and addressing OLWD’s injuries at home, self-care, and access to formal services, which should be essential components of FRM interventions. Care partners’ fatalistic perspectives could also provide an opportunity for care providers to develop a contingency plan with OLWD and their care partners for the possibility of a fall in order to prevent the health complications associated with lying on the floor after a fall [70]. Future studies should further investigate if and when care partners’ fatalistic perspective accurately reflects OLWD’s fall risk level, and how it might impact care partners’ FRM behaviours and OLWD’s fall risk, freedom of movement, autonomy, and quality of life.

## Limitations

The study has several limitations. First, because of the exploratory nature of this investigation, the findings may have limited generalizability since the study involved a small sample, which was recruited in a country in the Global North, majority non-Hispanic white, 60 years and older, spouse/partners and adult children, women, and college-educated. Prior studies have suggested that the gender of care partners might impact their FRM strategies; for example, one study showed sons caring for their mothers took only ‘protective’ and ‘coercive’ actions while daughters who were caring for their parents undertook “engaging” or “negotiating” actions [71]. Care partners of older adults from different countries and racial/ethnic groups might have different perceptions of fall risk and adopt different FRM behaviours [72]. For example, a study in Thailand described how the culture that stressed the high status of older people became a barrier for adult children to engage their parents who lived with dementia to exercise [73]. The framework presented here might not sufficiently reflect FRM behaviours adopted by care partners who were not women and those from different countries and racial/ethnic groups; therefore, it requires further refinement and validation with more diverse samples of care partners.

In addition, this study did not include secondary care partners. While we collected data about secondary care partners from OLWD’s primary care partners, the reports from the primary care partners might misrepresent the nature of the involvement of secondary care partners. Future work should include care partners from each of these roles. Furthermore, this study could not assess the efficacy of FRM strategies employed by care partners, nor could it identify any adverse events that may have resulted from FRM behaviours, such as mobility restraint, exercise support, or medication management. Future research needs to be done to assess dementia care partners’ FRM behaviours and the longitudinal impact of these behaviours on OLWD’s fall risk-related outcomes, autonomy, and quality of life and identify possible adverse events.

Despite these limitations, this study was the first study to systematically examine behaviours dementia care partners adopted to manage fall risk for community-dwelling OLWD. Different techniques of the Informed Grounded Theory approach were used to increase the credibility and the consistency of the findings. This study was the first step towards an empirically derived behavioural framework of care partners’ FRM for future validation and intervention development.

## Implications

Our findings have important implications for research, clinical practice, and policy. We proposed a behavioural framework that contributed to a better understanding of different roles care partners took when managing fall risk for OLWD living at home. We hope that this framework will allow future research to investigate associations between these behaviours and actual fall risk reduction and explore mechanisms of behavioural change for care partners to initiate, maintain, and modify their behaviours.

This behavioural framework, once validated, can be used to develop assessment tools for social and health service providers to examine care partners’ engagement in FRM while assessing OLWD’s risk of falling. Furthermore, these findings will inform future FRM intervention and policy development and implementation for managing fall risk for community-dwelling OLWD and their care partners. Social and health service providers can adopt multi-component interventions to strengthen care partners’ FRM behaviours that need to be enhanced, modify behaviours that might not be beneficial for OLWD, and mitigate the negative impact of these behaviours on health and well-being outcomes of OLWD and their care partners. These findings also have the potential to guide the development of technology-based interventions, such as mobile health apps [74], telemedicine [59], virtual fall management programs [75], and artificial intelligence [76], for improving care partners’ learning and ability to manage OLWD’s fall risk across different stages while maintaining their own health and well-being.

## Conclusion

This study utilized the Informed Grounded Theory approach to generate a new, comprehensive behavioural framework for conceptualizing the role of care partners in managing fall risk for OLWD. It entails four unique contributions: it identifies different domains of FRM behaviours in dementia care, provides a preliminary understanding of the divergent roles of primary and secondary care partners, describes behaviours adopted both with the purpose of reducing FRM and those adopted without this purpose but with potential impact on FRM, and captures four stages of FRM. This framework can be used to guide research, and upon validation, clinical care and intervention development aimed to reduce the disproportionately high and consequential fall risk of community-dwelling OLWD. This empirically derived behavioural framework of care partners’ FRM is a critical step for future intervention development to mitigate the negative impact of fall risk on OLWD, their care partners, and care systems.

## Supplementary Information

**Additional file 1.**

**Additional file 2.**

## Data Availability

The interview data generated during and/or analysed during the current study are not publicly available due to their sensitive personal nature and the possibility of revealing the identity of the participants. However, they are available from the corresponding author upon reasonable request.

## Abbreviations

OLWD: Older adults living with dementia
FRM: Fall risk management
CP: Care partners
NR: Not reported
