# Supplementary material for: Development of a behavioural framework for dementia care partners’ fall risk management
Source: BMC Geriatr. 2022 Dec 17;22:975. doi: 10.1186/s12877-022-03620-4 (PMC9758825; doi:10.1186/s12877-022-03620-4)
Supplement: Supplementary file 1 — Additional file 1. [file 12877_2022_3620_MOESM1_ESM.docx]

**Additional file 1.**

**Dementia care partners’ multi-level efforts in fall risk management**

| **Individual level efforts for elders with dementia**  · Keep elders company  · Increase surveillance and restrictions on elders  · Provide health promotion support for elders, including encourage exercise, promote use of walking aid, diet support  · Communicate with elders about fall risk |
| --- |
| **Interpersonal level efforts for elders with dementia**  · Change living arrangement  · Social withdrawal |
| **Physical environment level efforts for elders with dementia**  · Install assistive technology  · Modify home environment |
| **Community/institutional level efforts for elders with dementia**  · Get trained in exercise support, managing elders’ behaviors and stress, identifying home hazards  · Link elders with other care services resources, including purchasing fall risk-related equipment, decision-making in adopting fall risk-related strategies, providing consent on behalf of elders in participating programs, finding alternative help, accessing social services, escorting elders to care facilities  · Collaborate with professional health care team members: provide health information about elders, participate in making care plan, advocate for elders |

**Citation:**

[Zhou, Y., Strayer, A., Elizabeth, P., Sadak, T., & Hooyman, N. (2020). A mixed methods systematic review of informal caregivers’ experiences of fall risk among community-dwelling elders with dementia. *Health & Social Care in the Community*](https://www.zotero.org/google-docs/?wZGfB1). doi: [10.1111/hsc.13148](https://doi.org/10.1111/hsc.13148)
