# Supplementary material for: Development of a behavioural framework for dementia care partners’ fall risk management
Source: BMC Geriatr. 2022 Dec 17;22:975. doi: 10.1186/s12877-022-03620-4 (PMC9758825; doi:10.1186/s12877-022-03620-4)
Supplement: Supplementary file 2 — Additional file 2. [file 12877_2022_3620_MOESM2_ESM.docx]

**Additional file 2. Interview guide**

1. ***General experience of the caring process:***
2. **Can you tell me what supports you are providing to the OLWD (typical day)? How did you start to do these things?**
3. **How did you know about dementia or cognitive impairment of the OLWD?**
4. **Any other people are involved? What do others do for the OLWD?**
5. **Do you find the caring process challenging? Or stressed?**
   1. What are the main challenges?
6. **What are the main difficulties in providing this support?**
   1. What did you think? How did you address them?
   2. What helped you learn how to address those challenges?
7. **During your caring process, what are the things that worry you?**
8. ***Experiences of managing the OLWD’s risk of falling:***

*Note:* if this CP has more than one OLWD, discuss them one by one, trying to cover all of them if time permits.

1. **Have you thought about the risk of falling for the OLWD?**

**If the answer is yes:**

***I will first ask you about the first time you experienced the risk of falling for the OLWD.***

- 1. **What first brought this to you?**
  2. **Could you tell me about your thoughts and feelings then?**
     1. What did you know about fall risk in the OLWD then (factors, consequences)? How?
     2. How did you feel about it?
     3. How did fall risk in the OLWD impact the OLWD’s and your life?
  3. **Did you do anything to address the fall risk at that time?**
     1. If yes, what did you do?
     2. How did you learn about (what helped you) doing these things?
     3. Do you think those efforts -
        1. Reduced CP’s fall risk? How?
        2. Helped with their fear of falling? How?
        3. Improved their quality of life? Which aspects? How?
        4. Helped yourselves? How?
        5. What other changes do you think your efforts have made?
     4. If none above, why do you think that is?

***After the first time you realized the risk of falling for the OLWD, what happened ever since then.***

- 1. **What is your current understanding of the OLWD’s fall risk?**
     1. What do you know about fall risk in the OLWD now (factors, consequences)? How?
     2. How do you feel about it?
     3. How does fall risk in the OLWD impact the OLWD’s and your life?
  2. **Are you doing anything to address the fall risk for the OLWD ever since then?**
     1. If yes, what did you do?
     2. How did you learn about (what helped you) doing these things?
     3. Do you think those efforts -
        1. Reduced the OLWD’s fall risk? How?
        2. Helped with their fear of falling? How?
        3. Improved their quality of life? Which aspects? How?
        4. Helped yourselves? How?
        5. What other changes do you think your efforts have made?
     4. If none above, why do you think that is?

***Now I want to ask questions about how other people might get involved in providing support for the OLWD.***

- 1. **Does someone else that the OLWD knows mention the risk of falling?**
     1. If yes, what did they do?
     2. How did they learn about doing these things based on your observation?
     3. Do you think their efforts -
        1. Reduced the OLWD’s fall risk? How?
        2. Helped with their fear of falling? How?
        3. Improved their quality of life? Which aspects? How?
        4. Helped yourselves? How?
        5. What other changes do you think your efforts have made?
     4. If none above, why do you think that is?

***If the answer is no***

- 1. **Have the OLWD ever fallen before?**
  2. **Has anyone talked to you about the risk of falling before? Did they do anything about it?**
     1. If at least one yes for questions g and h, what happened at that time and what did you think about that?
     2. If no both for g and h, ask: you said you did not think about the OLWD’s risk of falling, why do you think that is?

1. **Is there anything you have done that you believe is helpful for addressing fall risk for the OLWD? What are they? Why do you think it’s helpful? How did you learn about this?**
2. **Have you ever provided any following support to OLWD?**

| **CP behaviours** | **Y/N** | **Facilitators/ barriers** | **Impact on OLWD’s health outcomes** | **Impact on CP’s health outcomes** |
| --- | --- | --- | --- | --- |
| Increase surveillance and restrictions on the OLWD |  |  |  |  |
| Physical exercise |  |  |  |  |
| Home safety modification |  |  |  |  |
| Improve the OLWD’s nutrition |  |  |  |  |
| Encourage use of walking aids |  |  |  |  |
| Install assistant technology |  |  |  |  |
| Communicate with the OLWD about their fall risk or their risky behaviours |  |  |  |  |
| Seek information, participate in any programs, or receiving social and health services |  |  |  |  |
| Change your own behaviours or life arrangement |  |  |  |  |
| Receive help from other care partners * |  |  |  |  |
| Mobility assistance * |  |  |  |  |
| Assess and address the OLWD’s health conditions * |  |  |  |  |
| Supporting OLWD’s help-seeking behaviours * |  |  |  |  |

*Behaviours with * were added based on the preliminary analysis of the interviews with the first five participants.

1. **What might be missing from this checklist?**
2. **Do you plan to continue or to try anything, in order to address the fall risk and these challenges?**
   1. Why or why not?
   2. What do you want to do?
   3. What is your hope? Why?
3. **Is there anything that you think could help you address the OLWD’s risk of falling and the challenges? (It could be any services, intervention, information, extra support, technology, etc.) Will you try to get them? Why or why not?**
4. **What do you think are the most important things to do for the OLWD in the face of their fall risk? Why do you think these are important?**
5. **Is there anything that you want to add?**
6. **We are still in the phase of piloting these questions and improving the interview process. Do you have any feedback about the interview process?**

*Note*: This question was asked during the first five interviews.
